# Growth Mechanism of SmB_6_ Nanowires Synthesized by Chemical Vapor Deposition: Catalyst-Assisted and Catalyst-Free

**DOI:** 10.3390/nano9081062

**Published:** 2019-07-24

**Authors:** Yi Chu, Yugui Cui, Shaoyun Huang, Yingjie Xing, Hongqi Xu

**Affiliations:** Beijing Key Laboratory of Quantum Devices, Key Laboratory for the Physics and Chemistry of Nanodevices, and Department of Electronics, Peking University, Beijing 100871, China

**Keywords:** nanowires, synthesis, CVD, topological insulators, growth mechanism

## Abstract

SmB_6_ nanowires, as a prototype of nanostructured topological Kondo insulator, have shown rich novel physical phenomena relating to their surface. Catalyst-assisted chemical vapor deposition (CVD) is a common approach to prepare SmB_6_ nanowires and Ni is the most popular catalyst used to initiate the growth of SmB_6_ nanowires. Here, we study the effect of growth mechanism on the surface of SmB_6_ nanowires synthesized by CVD. Two types of SmB_6_ nanowires are obtained when using Ni as the catalyst. In addition to pure SmB_6_ nanowires without Ni impurity, a small amount of Ni is detected on the surface of some SmB_6_ nanowires by element analysis with transmission electron microscopy. In order to eliminate the possible distribution of Ni on nanowire surface, we synthesize single crystalline SmB_6_ nanowires by CVD without using catalyst. The difference between catalyst-assisted and catalyst-free growth mechanism is discussed.

## 1. Introduction

Topological insulator has attracted much interest in the field of condensed matter physics and material science recently because of its fundamentally novel physical phenomena, such as nontrivial gapless surface states protected by time reversal symmetry [1,2,3]. Topological Kondo insulator extends the family of topological insulators by the intersection of topological insulator and heavy fermion (Kondo) compound. Theoretical and experimental efforts have shown that topological Kondo insulator is a promising building block for realizing spintronics and majorana fermions devices in future [4,5]. SmB_6_ is a well-known Kondo insulator and has been identified as a prototype of topological Kondo insulator [6]. Magnetotransport measurement have revealed topological insulator properties in SmB_6_, such as spin-polarized surface state transport and linear positive magnetoresistance at low field [7,8,9].

One-dimensional SmB_6_ nanostructure is suitable to investigate the physical phenomenon relating to the surface due to its large aspect ratio. For example, higher aspect ratio is proposed to be the reason for bigger activation energy in SmB_6_ nanobelt [10]. He et al. report strong surface magnetism and hysteretic magnetoresistance in thin SmB_6_ nanowires [11]. A size-dependent surface magnetism is concluded in thin SmB_6_ nanowire independent of the strong correlations and spin-orbit interactions. The exposed surface of SmB_6_ nanowire seems to show more novel behavior than that of the bulk crystal. In order to further understand the relation between novel physical phenomena and the surface of SmB_6_ nanowires, more investigation of the growth of SmB_6_ nanowires is highly desirable.

SmB_6_ nanowires have been synthesized by heating Sm with BCl_3_ in the ambient of H_2_ and Ar [12]. However, the surface of SmB_6_ nanowires prepared in this way is covered by a layer with the thickness of ~10 nm, which is not suitable to evaluate their surface conduction. In another experiment without catalyst on the substrate, rarely growth of SmB_6_ nanowire is found [8]. SmB_6_ nanowires are usually prepared by chemical vapor deposition (CVD) with metal catalyst, e.g., Ni and Pd [10,13,14]. Vapor-liquid-solid (VLS) mechanism is usually employed to explain the growth of SmB_6_ nanowires with the help of catalyst. However, VLS growth has an intrinsic disadvantage, which is the possible migration of catalyst along the nanowire sidewall during the nanowire growth under some conditions. High resolution transmission electron microscopy (HRTEM) have observed gold or aluminum catalyst on the surface of Si nanowires [15,16,17,18]. Since the transport of semiconductor nanowire is mainly decided by the bulk of nanowire, the occasional distribution of trace amount of catalyst on nanowire sidewall is rarely considered in the study of semiconductor nanowire. Nonetheless, the influence of surface impurity on topological insulator cannot be ignored simply. If Ni, the most popular catalyst assisting the growth of boride nanowire [10,13,19,20,21], migrates along the nanowire surface, the time reversal symmetry in topological Kondo insulator SmB_6_ can be broken [1,22,23]. Such a problem has not been emphasized and studied in SmB_6_ nanowires.

In this work, we prepare SmB_6_ nanowires by CVD under two conditions: with and without Ni catalyst. The structure and element composition of SmB_6_ nanowires grown with Ni catalyst are analyzed by HRTEM in detail and Ni is found on the surface of some SmB_6_ nanowires. This observation reveals that Ni catalyst may migrate on the surface of SmB_6_ nanowires during the growth period. In order to eliminate the possible appearance of catalyst impurity on nanowire surface, we prepare SmB_6_ nanowires by a novel method without catalyst. Single crystalline SmB_6_ nanowires are grown on SmB_6_ particles via vapor-solid (VS) mechanism.

## 2. Materials and Methods 

Small Si plates are used as the substrate. Samarium chloride (SmCl_3_, purity 99.99%, ALADDIN, China) is used as the source of samarium. The mixture of boron and boron trioxide powder (weight ratio 1:1, purity 99.99%, CNM, China) is heated to generate gaseous B_2_O_2_. SmB_6_ nanowires are grown under two conditions: with and without Ni catalyst. A thin Ni film with the thickness of 10 nm is deposited on Si plate as the catalyst for nanowire growth in some experiments. In other experiments without catalyst, commercial SmB_6_ particles washed by hydrochloric acid are dispersed on bare Si plate to help the growth of nanowires. In all experiments, the source and the substrate are placed in a quartz tube which is heated in a tube furnace. The heating temperature of the tune furnace is 1100 °C and the heating time is 1–2 h. A mixture of Argon (285 sccm) and hydrogen (30 sccm) is used as the carrier gas.

The morphology of nanowires is observed by scanning electron microscope (SEM, FEI Quanta 600, Hillsboro, OR, USA). The highly magnified morphology, crystal structure and the element composition are analyzed by high resolution transmission electron microscopes (HRTEM, FEI Tecnai F20, Hillsboro, OR, USA) equipped with selected area electron diffraction (SAED) and energy dispersive X-ray spectroscopy (EDAX). Some nanowires are analyzed by HRTEM (FEI Tecnai F30, Hillsboro, OR, USA) equipped with electron energy loss spectroscopy (EELS). The structure of as-grown nanowires is determined by X-ray diffraction (XRD, PANalytical X-Pert3, Almelo, Netherlands). Raman spectrum of as-grown nanowires is analyzed by powder Raman spectroscopy (Horiba Jobin-Yvon LabRAM HR800, Kyoto, Japan). Nanodevices based on nanowires grown with and without Ni catalyst are fabricated and some measurement result is shown in the Supplementary Materials.

## 3. Results and Discussion

**Figure 1:**
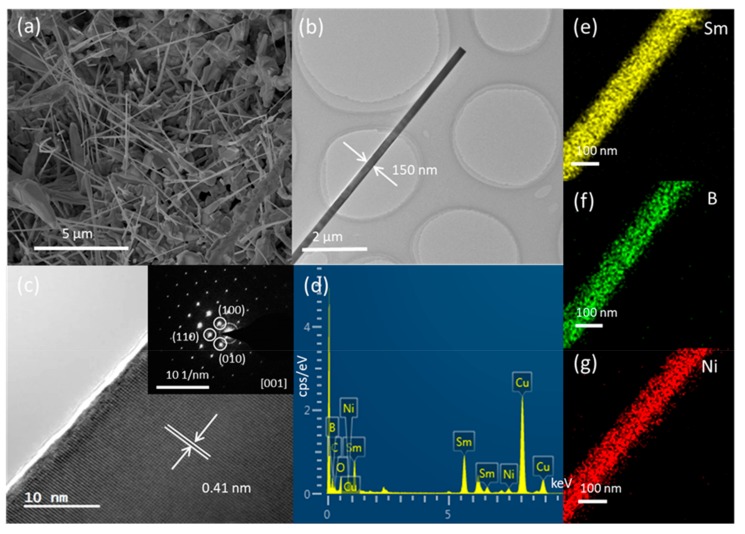
SmB_6_ nanowires grown on Ni coated Si substrate. (**a**) SEM image; (**b**) TEM image, (**c**) HRTEM image and (**d**) EDAX spectrum of a single nanowire; element mapping of Sm (**e**), B (**f**), and Ni (**g**) of a single nanowire.

First, the growth of SmB_6_ nanowires is realized on Ni-coated Si substrate in a way similar to the method reported in Ref. 10. Straight nanowires grown on the substrate are shown in Figure 1a. The nanowires have a length of several microns and a diameter of ~100 nm. Several tens of nanowires are observed by TEM and nine nanowires are analyzed in detail. No defect is observed in all nanowires. Figure 1b shows a typical TEM image of a single nanowire. The structure of this nanowire is shown in Figure 1c and the corresponding SAED pattern is shown in the inset of Figure 1c. Both the crystal structure and diffraction pattern are in accordance with SmB_6_. (001) fringes with d-spacing of 0.41 nm are observed. The composition of nanowire is analyzed by EDAX. Sm, B, Cu, C, O, and Ni are detected in nanowire (as shown in Figure 1d). C and Cu come from the carbon film covered copper grid. Oxygen may come from the carbon film and/or the thin sheath of the nanowire. Figure 1e and 1f show the element mapping image of Sm and B, respectively. Uniform distribution of Sm and B is observed in the nanowire. The atomic ratio of Sm:B in this nanowire is approximately 1:6. Among all samples investigated by EDAX, the largest atomic ratio of Sm:B is ~1:7. Such a deviated stoichiometry may be caused by the large difference between low atomic number of boron and high atomic number of Sm [14]. Above characterization reveals that single crystalline SmB_6_ nanowires are grown by CVD with the help of Ni. Although no particle is found at the tip of SmB_6_ nanowire (as shown in Figure 1a and 1b), we still believe the growth of SmB_6_ nanowires is controlled by VLS mechanism. This phenomenon is similar to SmB_6_ nanobelts grown via a base growth model [10]. More details of VLS growth process will be discussed below.

**Figure 2:**
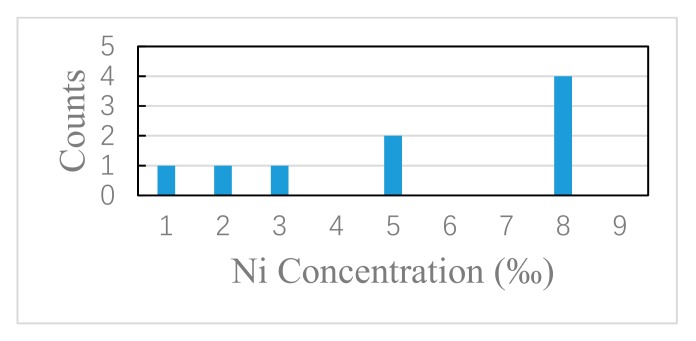
Ni concentration in nine SmB_6_ nanowires.

We carefully check the concentration of Ni in SmB_6_ nanowire by EDAX. Different concentration is detected in nanowires with similar morphology. Ni concentration varies in a range of 0.1–0.8 atomic percent in this work. The element mapping of Ni with the concentration of 0.8% is shown in Figure 1g. Figure 2 shows Ni concentration in nine measured nanowires. It should be noted that the trace amount of Ni in EDAX spectrum does not definitely indicate the existence of Ni in nanowire because of the background noise of detection signal in EDAX. In the TEM we used, 0.5 atomic percent is regarded as the threshold of noise signal collection, and only those values larger than 0.5% are considered to reflect the signals from the nanowire without doubt. Among nine nanowires, Ni concentration of 0.8% is detected in four nanowires. The average Ni concentration is 0.32% for the other five nanowires, which roughly reflects the level of the background noise. The concentration gap between the average value (0.32%) and the conceivable collection (0.8%) clearly divides nanowires into two categories: with and without Ni impurity. The actual position of Ni impurity in the nanowire cannot be unequivocally determined by EDAX due to the weak signal level of Ni. There are two possible locations for Ni impurity: Bulk and surface. We think that if Ni bulk doping occurs during the period of nanowire growth, the measured concentration of Ni in SmB_6_ nanowire should roughly obey Gaussian distribution. The histogram in Figure 2 obviously deviates from the shape of Gaussian distribution, which indicates the other position: on the nanowire surface. We know that catalyst migration along the nanowire sidewall during VLS growth can bring catalyst to the surface [15,16,17,18]. Since there is no other Ni source than Ni film on the substrate, we believe the migration of Ni catalyst from the bottom occur in some nanowires. This growth process is similar to the base growth of alkaline-earth metal hexaboride nanowires when Ni is used as the catalyst [20].

Magnetic atom shows particularly important effect on the surface transport in topological insulator [1,22,23]. The appearance of Ni on nanowire surface may deteriorate the nontrivial surface states protected by time reversal symmetry. Other method than VLS growth is desirable in order to eliminate the influence of Ni catalyst. Vapor-solid mechanism has been used to form various semiconductor nanowires. Anisotropic growth starting from crystal nucleus and/or substrate-mediated diffusion can induce one-dimensional growth [24,25,26]. However, it seems rather difficult to grow SmB_6_ nanowire via VS mechanism. The necessity of Ni catalyst on the growth of boride nanowires is emphasized even when no particle is found at the tip of nanowire [10,20]. Only sparse SmB_6_ nanowires are found randomly at structure defect sites on the edge of Si substrate in literature [8].

**Figure 3:**
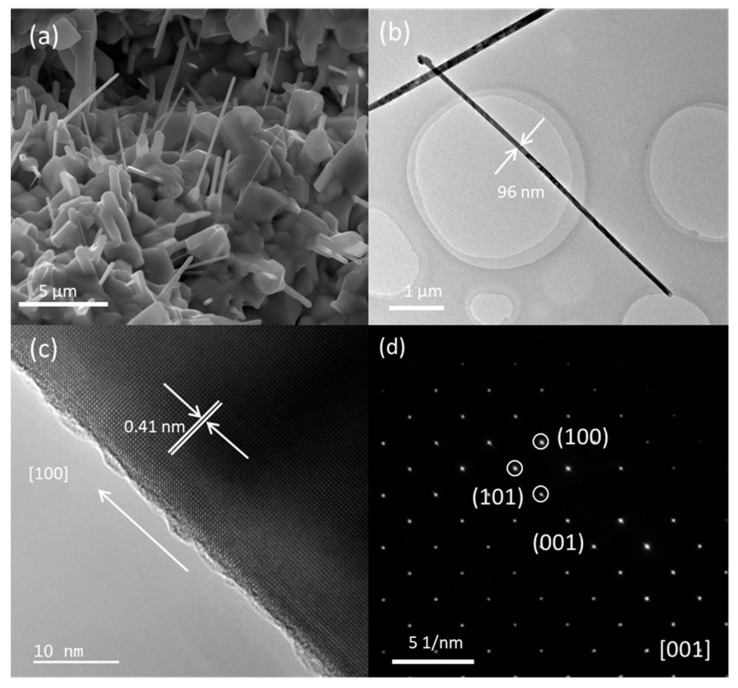
SmB_6_ nanowires grown on SmB_6_ particles dispersed on Si substrate. (**a**) SEM image of SmB6 nanowires; (**b**) TEM image, (**c**) HRTEM image and (**d**) SAED pattern of a single nanowire.

We improve the growth of SmB_6_ nanowires by dispersing SmB_6_ particles on bare Si plate. No catalyst is used to initiate the growth of SmB_6_ nanowires in this case. Figure 3a shows SmB_6_ nanowires grown on SmB_6_ particles. The nanowires in Figure 3a are a little shorter and in less density comparing to the nanowires in Figure 1a. As expected, no particle is observed at the tip of SmB_6_ nanowire prepared under this condition. Figure 3b shows a TEM image of a single nanowire. HRTEM image and SAED pattern of this nanowire are shown in Figure 3c,d and are in accordance with the structure of SmB_6_. Element mapping of this nanowire shows uniform distribution of Sm and B. The concentration of Ni is 0.4% in this nanowire, which is below the threshold value of the instrument and is similar to the base level of pure SmB_6_ nanowires grown via VLS mechanism (0.32%). Above result shows single crystal SmB_6_ nanowires are synthesized without Ni catalyst in large scale. Obviously, the growth of SmB_6_ nanowires in this experiment is controlled by VS mechanism. [24,26] We believe that SmB_6_ particles contribute suitable nucleation sites for the formation of SmB_6_ nuclei, resulting in the growth of SmB_6_ nanowires without metal catalyst.

**Figure 4:**
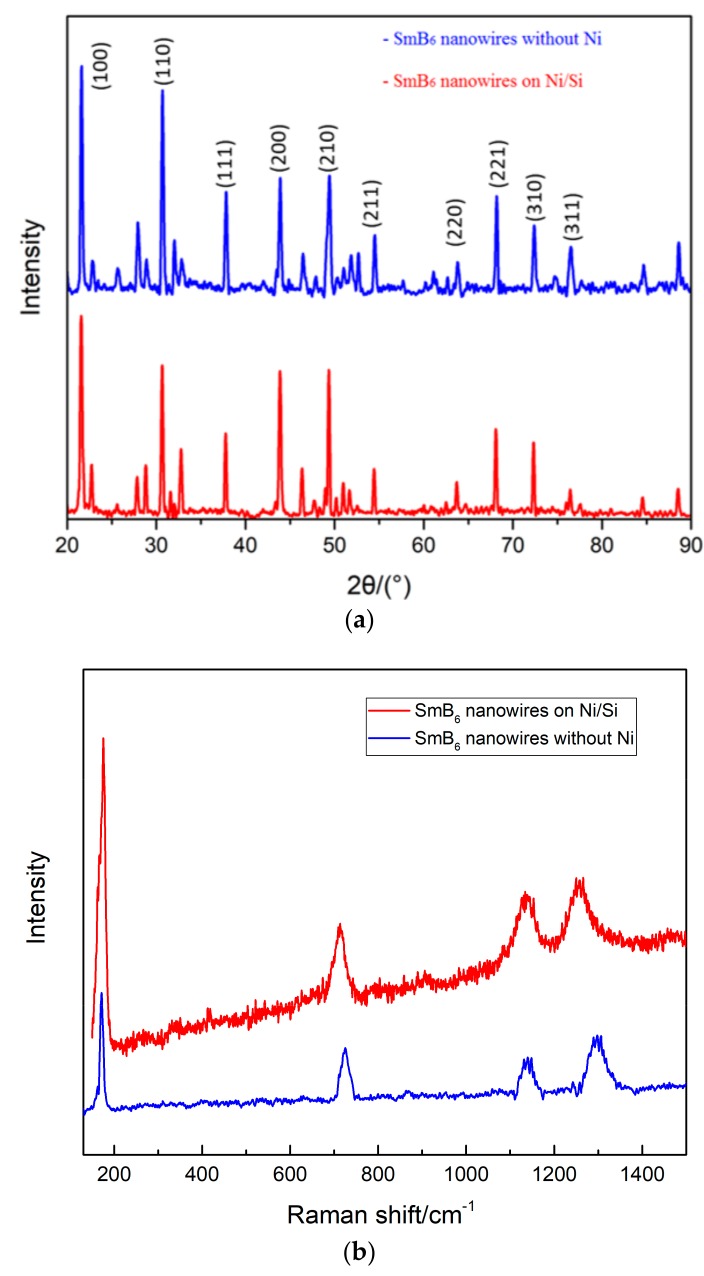
(**a**) XRD spectrum of SmB_6_ nanowires grown with Ni catalyst, (**b**) Raman spectrum of SmB6 nanowires grown with Ni (top) and without Ni (bottom).

Other analysis techniques than HRTEM are employed to analyze the structure and optical property of SmB_6_ nanowires. We check the nanowires grown with and without Ni catalyst by EELS. Same conclusion of Ni impurity is obtained in EELS measurement as above EDAX detection. As-grown nanowires on Si substrate are measured by XRD. Figure 4a shows XRD spectrum of SmB_6_ nanowires grown with and without Ni catalyst. All strong peaks are well assigned to the structure of SmB_6_. We note that the XRD peaks in the sample of nanowires grown on particles contains the contribution of both nanowires and particles. XRD analysis cannot distinguish nanowires and particles. Figure 4b shows Raman spectrum of SmB_6_ nanowires measured in air. The sharp peak at 174 cm^−1^ should come from the infrared active T_1u_ mode. Three broad peaks at 724, 1147, and 1279 cm^−1^ are also detected in both samples and are indexed to A_1g_, E_g_, and T_2g_ phonon modes of SmB_6_ [8,10]. Raman measurement shows no difference in two samples. We also measure Raman spectrum of pure SmB_6_ particles dispersed on Si plate and no obvious peak is detected. A strong scattering is proposed for the lack of Raman peak in this case. Therefore, nanowires grown on particles have an important contribution to the bottom Raman spectrum in Figure 4b.

**Figure 5:**
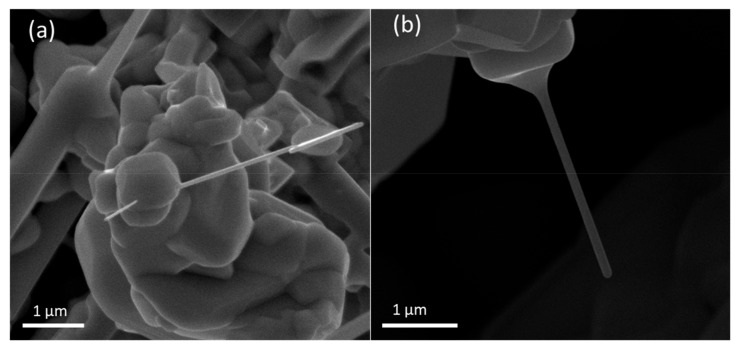
SEM image of SmB6 nanowire grown on (**a**) irregular particle and (**b**) cubic block.

Although the growth of SmB_6_ nanowires has been achieved with the help of different catalyst in literature, there are still some puzzles about the role of catalyst in nanowire growth. For example, in the spherical tip of SmB_6_ nanowires grown with Pd catalyst, neither Sm nor B but PdSi_x_ alloy is found [14]. A different morphology appears when Au is used as the catalyst. A large number of SmB_6_ nanowires form on gold coated substrate but no particle is observed at the tip of nanowire [8]. Even for Ni, spherical tip is just observed in some nanowires, whereas for other nanobelts formed in the same batch, no tip is found [10]. All these phenomena indicate that the growth process of SmB_6_ nanowires is quite sensitive to the experimental condition. SmB_6_ nanowires in Figure 1a are prepared by a similar procedure and show similar morphology as those nanobelts in Ref. 10, suggesting a similar unconventional VLS growth mechanism with Ni catalyst. The Ni-assisted growth model of SmB_6_ nanowires in our experiment can be briefly described as following. Sm and B in the vapor phase dissolve in liquid droplets continuously after the liquid droplet forms on the substrate, and SmB_6_ nanocrystal will precipitate out from the droplets after the droplet becomes supersaturated. If the concentration of Sm and B in the atmosphere is high enough, some vapor source may form a solid shell surrounding the droplet during the period of nanowire growth. Other than spherical particle in traditional VLS mechanism, irregular particles are formed beneath nanowires in this base growth process [10]. A SEM image of the root of a SmB_6_ nanowire is shown in Figure 5a. Irregular particle at the bottom of nanowire is consistent with this growth model. As a side effect of such base growth, Ni catalyst may diffuse upwards along the sidewalls of SmB_6_ nanowires, which is similar to gold diffusion on Si nanowire surface in literature [15,17,18]. Since the diffusion of catalyst sensitively relates to the thermodynamic and kinetic factors decided by the interface between liquid bottom droplet and solid nanowire [16], two categories of SmB_6_ nanowires, with and without Ni on the surface, are formed in the same batch in our experiment. Our analysis shows that even with microscopy characterization like TEM-EDAX, it is still difficult to determine whether the migration of Ni catalyst occurs or not. In order to obtain a reliable result of surface conduction and magnetism in VLS grown SmB_6_ nanowire, we suggest that combination of transport measurement and TEM-EDAX observation of a same single nanowire is needed in future, because different Ni concentration may occur in different nanowires, e.g., nanowire A shows a negligible Ni concentration in TEM-EDAX analysis but nanowire B measured by magnetotransport has a higher Ni concentration.

VS growth of SmB_6_ nanowires without any catalyst is achieved on SmB_6_ particles in this work. A SmB_6_ nanowire grown on a cubic SmB_6_ block is shown in Figure 5b. It is not clear whether epitaxy occurs in this case or not. Comparing to the nanowires grown with Ni catalyst, same structure and morphology are observed in SmB_6_ nanowires grown without Ni catalyst. This fact means that same procedure of device fabrication is adequate to these two kinds of nanowires. However, considering no Ni is included in VS growth, we think SmB_6_ nanowires grown via VS mechanism are a better platform for investigation of rich physical properties of SmB_6_, such as surface conduction and magnetism. A problem of VS growth is smaller density of SmB_6_ nanowires sometimes. This phenomenon means that even on SmB_6_ particle, the formation of solid SmB_6_ nuclei is still less efficient than the generation of catalyst liquid droplet by alloying. We think that better treatment of SmB_6_ particles may provide more suitable sites for nuclei formation.

A disadvantage of this work is the uncontrollable distribution and growth orientation of SmB_6_ nanowires. Although IV and III–V nanowires grown under some conditions show governed distribution and growth orientation [24], it seems that the growth of SmB_6_ nanowires by CVD, both in present work and in literature, is very difficult to control the nanowire distribution and growth orientation dedicatedly, possibly because of the difficulty of providing two independent sources (Sm and B) properly and simultaneously. Figure 5 shows the important role of the particle at root of nanowire, either with Ni catalyst or without catalyst. Epitaxy growth on SmB_6_ single crystal wafer, which may help to form the bottom particles regularly, is a possible way to improve the distribution and growth orientation of SmB_6_ nanowires.

We propose that more analysis and understanding of the growth process may bring novel magnetotransport result in SmB_6_ nanowire grown with Ni catalyst. Ni atoms on the surface of VLS grown SmB_6_ nanowires can break the time reversal symmetry and lead to a gap in the surface states. In VLS growth process of SmB_6_ nanowires, if the migration of Ni can be restricted or initiated by some condition, the gap in the surface states can be selectively closed or opened. Dirac electrons can be confined by the gap and novel phenomena of quantized mode of Dirac electronic surface states may be observed. This device may contribute a new route other than the etching technique towards topological insulator quantum dot [27].

## 4. Conclusions

SmB_6_ nanowires are prepared by CVD under two conditions: With and without Ni catalyst. Ni is found on the surface of some SmB_6_ nanowires grown with Ni catalyst. The growth model of SmB_6_ nanowires with Ni catalyst is concluded to be base grown VLS mechanism. VS mechanism is used to prepare SmB_6_ nanowires without Ni catalyst. No other difference than surface impurity is observed in SmB_6_ nanowires grown with and without Ni catalyst.

## Supplementary Materials

The following are available online at https://www.mdpi.com/2079-4991/9/8/1062/s1, Figure S1: Schematic device structure; Figure S2: SEM image of fabricated device; Figure S3: I-V plot measured in a probe station.
