# Supplementary material for: Growth Mechanism of SmB6 Nanowires Synthesized by Chemical Vapor Deposition: Catalyst-Assisted and Catalyst-Free
Source: Nanomaterials (Basel). 2019 Jul 24;9(8):1062. doi: 10.3390/nano9081062 (PMC6722856; doi:10.3390/nano9081062)
Supplement: Supplementary file 1 [file nanomaterials-09-01062-s001.pdf]

Supplementary information

Growth mechanism of SmB_6 nanowires synthesized by chemical vapor deposition: catalyst-assisted and catalyst-free

Yi Chu, Yugui Cui, Shaoyun Huang, Yingjie Xing*, and H. Q. Xu

Beijing Key Laboratory of Quantum Devices, Key Laboratory for the Physics and Chemistry of Nanodevices, and Department of Electronics, Peking University, Beijing 100871, P. R. China

Device fabrication and measurement

We have fabricated and measured some devices based on nanowires grown with and without Ni catalyst (Figure 1). The nanowires are mechanically transferred from growth substrates onto a SiO_2(200 nm)/Si substrate with predefined positioning marks for device fabrication. Isolating nanowires are selected to define electrical contacts by electron beam lithography (EBL) process. An argon plasma clean process is performed to remove the residual EBL resist and native surface oxides in contact area. Then Ti(5 nm)/Au(270 nm) electrodes are deposited by sputter to complete the device (Figure 2). These devices are tested in a probe station. Some typical results of our devices are shown below. The I-V plot shows a high resistance (~ MΩ) in all fabricated devices (Figure 3), regardless of the nanowire type. Based on our experiences, we think such a large resistance means a high contact resistance between the electrode and the nanowire.

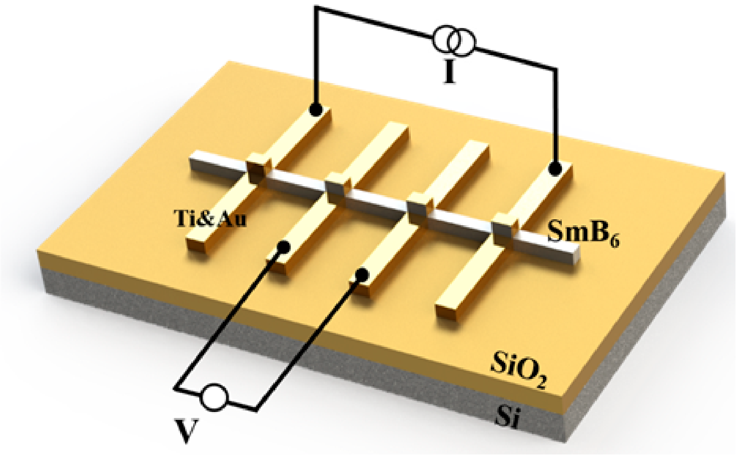

Figure 1 Schematic device structure.

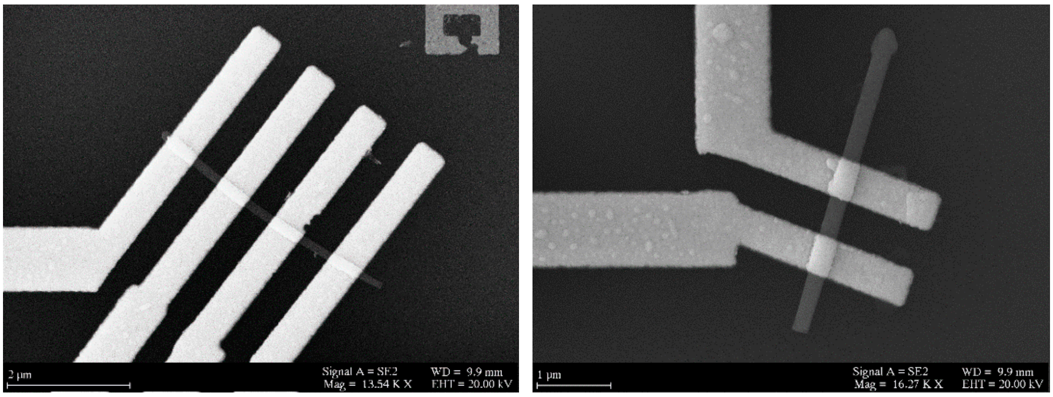

Figure 2 SEM image of fabricated device. (left): Four terminal; (right): two terminal.

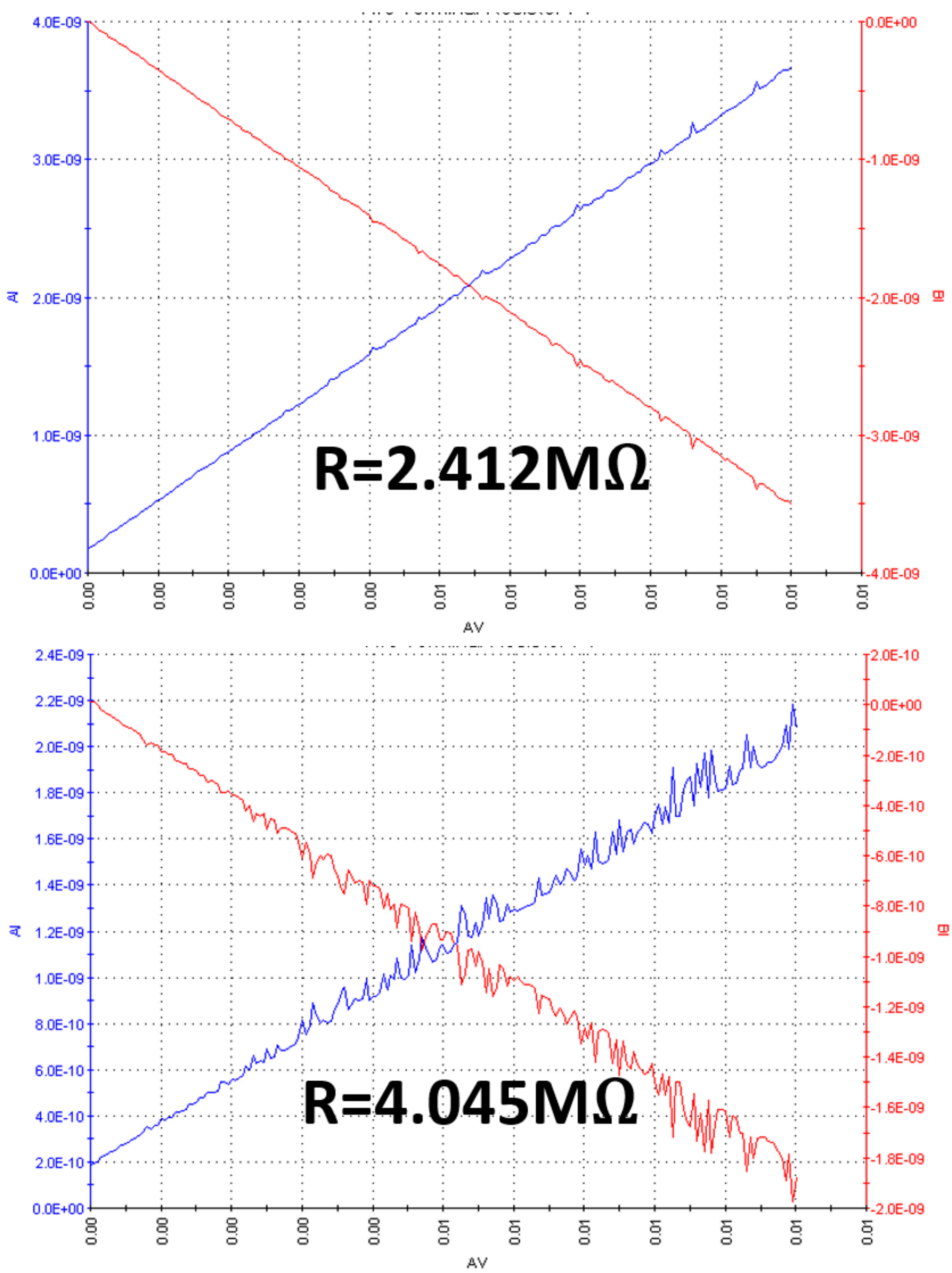

Figure 3. I-V plot measured in a probe station. (top): SmB_6 nanowire grown with Ni catalyst; (bottom) SmB_6 nanowire grown without Ni catalyst.
